# Expression of the Alternative Oxidase Influences Jun N-Terminal Kinase Signaling and Cell Migration

**DOI:** 10.1128/MCB.00110-18

**Published:** 2018-11-28

**Authors:** Ana Andjelković, Amelia Mordas, Lyon Bruinsma, Annika Ketola, Giuseppe Cannino, Luca Giordano, Praveen K. Dhandapani, Marten Szibor, Eric Dufour, Howard T. Jacobs

**Affiliations:** a Faculty of Medicine and Life Sciences, University of Tampere, Tampere, Finland; b BioMediTech Institute, University of Tampere, Tampere, Finland; c Institute of Biotechnology, University of Helsinki, Helsinki, Finland

**Keywords:** AP-1, Jun N-terminal kinase, alternative oxidase, transcription, wound healing

## Abstract

Downregulation of Jun N-terminal kinase (JNK) signaling inhibits cell migration in diverse model systems. In Drosophila pupal development, attenuated JNK signaling in the thoracic dorsal epithelium leads to defective midline closure, resulting in cleft thorax. Here we report that concomitant expression of the Ciona intestinalis alternative oxidase (AOX) was able to compensate for JNK pathway downregulation, substantially correcting the cleft thorax phenotype. AOX expression also promoted wound-healing behavior and single-cell migration in immortalized mouse embryonic fibroblasts (iMEFs), counteracting the effect of JNK pathway inhibition. However, AOX was not able to rescue developmental phenotypes resulting from knockdown of the AP-1 transcription factor, the canonical target of JNK, nor its targets and had no effect on AP-1-dependent transcription. The migration of AOX-expressing iMEFs in the wound-healing assay was differentially stimulated by antimycin A, which redirects respiratory electron flow through AOX, altering the balance between mitochondrial ATP and heat production. Since other treatments affecting mitochondrial ATP did not stimulate wound healing, we propose increased mitochondrial heat production as the most likely primary mechanism of action of AOX in promoting cell migration in these various contexts.

## Abstract (precis)

Downregulation of Jun N-terminal kinase (JNK) signaling inhibits cell migration in diverse model systems. In Drosophila pupal development, attenuated JNK signaling in the thoracic dorsal epithelium leads to defective midline closure, resulting in cleft thorax.

## INTRODUCTION

Cell migration is an essential process in animal development, as well as in tissue repair. It has been widely studied in model systems, where the focus has been largely on mechanosensation and mechanotransduction (1, 2). The transcriptional and cytoskeletal regulation of cell migration ensures coordination and an ability to respond to extrinsic and intrinsic cues (2). At the cellular level, the most studied mammalian model is the scratch or wound-healing assay, in which a linear scratch is made in a confluent monolayer of cells, which then migrate to close the gap at a measurable rate (3). In Drosophila development, cell migration has been studied in embryogenesis, in the process of dorsal closure (4, 5), and later on during metamorphosis, when many of the same genes are involved in thoracic closure (6). This process involves cells everting from the wing imaginal discs, which spread over the preexisting larval epidermis (7). These migrating cell sheets eventually fuse at the midline to create a closed epithelial layer that gives rise to the cuticular structures of the dorsal thorax.

In an earlier study (8), we reported that the process of dorsal thoracic closure is disrupted by the expression of a commonly used, inducible driver of transgene expression, GeneSwitch, in the presence of the inducing steroid RU486. GeneSwitch is a modified version of the Saccharomyces cerevisiae transcription factor GAL4 incorporating the ligand-binding domain of the progesterone receptor so as to place it under steroid control (9, 10). Since progesterone or its analogues are not found in Drosophila, it had been assumed that GeneSwitch plus RU486 would be phenotypically inert in otherwise wild-type flies, which is indeed the case in adults. Although cleft thorax was the most dramatic and frequent phenotype observed in GeneSwitch-expressing flies reared throughout development on RU486-containing medium, other developmental dysmorphologies were also observed, including wings with apoptotic regions, abnormal or missing bristles, and cleft abdomen.

In the course of these studies, we observed that coexpression of the mitochondrial alternative oxidase (AOX) from Ciona intestinalis was able to revert the cleft thorax and other dysmorphological phenotypes brought about by GeneSwitch plus RU486 (8). Expression of an otherwise inert transgene, such as green fluorescent protein (GFP), the alternative NADH dehydrogenase Ndi1 from yeast, or even a catalytically inactive variant of AOX, was unable to correct GeneSwitch-plus-RU486-induced cleft thorax (8).

AOX represents an accessory component of the mitochondrial respiratory chain (RC), which is found in microbes, plants, and some metazoan phyla but not insects or vertebrates (11). AOX provides a non-proton-motive bypass for complexes III (cIII) and IV (cIV) of the standard RC. In various contexts, it is able to relieve metabolically deleterious stresses arising from damage, toxic inhibition, or overload of the RC (11, 12). Furthermore, when expressed in human cells, flies, or mice, *Ciona* AOX can alleviate the damaging phenotypes associated with RC inhibition (13–19). However, the link between respiratory homeostasis and dysmorphologies resulting from GeneSwitch plus RU486 is unknown.

These findings prompted us to test whether AOX could revert the cleft thorax phenotype brought about by genetic manipulations in the signaling network that maintains the migratory behavior of the cell sheets everting from the wing discs. Three such classes of mutants have been studied. First, cleft thorax is manifested by specific, recessive alleles of the gene encoding the Drosophila RXR homologue, ultraspiracle (usp), which acts as a dimerization partner for the ecdysone receptor (20). Second, compound heterozygotes for another essential transcription factor, the GATA factor pannier (pnr), also give rise to this phenotype (21). One *pnr* allele used in these studies is *pnr^MD237^*, a hypomorph created by insertion of GAL4 into the promoter region for one of the two antagonistic pnr isoforms. This allele was originally isolated in an enhancer-trap screen and has proven useful as a driver of transgene expression in the specific domain of *pnr* expression in the dorsal epithelium; thus, it is often referred to as *pnr*-GAL4.

**FIG 1:**
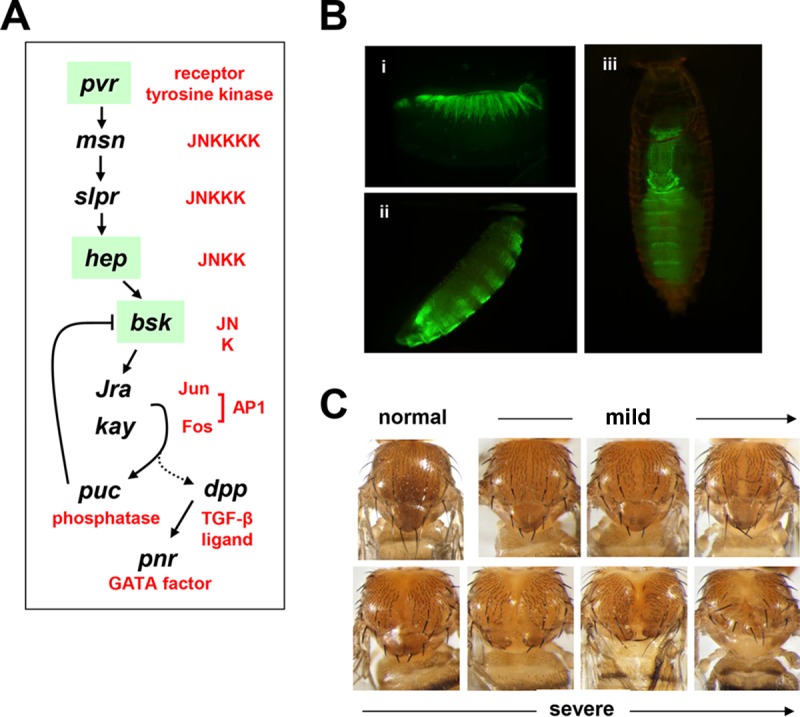
Cleft thorax produced by downregulation of JNK signaling. (A) Summary of the main steps in the JNK signaling cascade in Drosophila thoracic development indicating Drosophila genes by their standard symbols and their functional assignments in red text. The dotted line to *dpp* represents its activation by AP-1 in embryonic dorsal closure but not in pupal thoracic closure. *pnr* is activated by *dpp* to regulate the dorsal phenotype. The steps indicated with a green background are the ones that were clearly influenced by AOX, based on the data presented later in the paper. TGF-β, transforming growth factor β. (B) Live-cell imaging of a 13- to 15-h-old embryo (i), an L3-stage larva (ii), and a pupa (iii) of flies expressing GFP under the control of the *pnr*-GAL4 driver (original *pnr^MD237^* strain). (C) Examples of thoracic phenotypes scored as normal, mild, or severe, with arrows indicating the trend within each class toward more severe cleft thorax phenotypes.

Third, cleft thorax results from mutations in the Jun N-terminal kinase (JNK) signaling pathway (4) (Fig. 1A). JNK (22) is a member of the mitogen-activated protein (MAP) kinase family that activates the AP-1 transcription factor by phosphorylating its c-Jun subunit (23). AP-1 has a plethora of cellular roles, which include the regulation of cell migration both in development (24) and in pathology, e.g., tumor invasion (25). It is also subject to many types of regulation (26). JNK is itself activated by a variety of stresses through a classic kinase cascade (27, 28). In the context of thoracic closure, the initiating stimulus appears to be the engagement of receptor tyrosine kinase pvr (29) (PDGF [platelet-derived growth factor] and VEGF [vascular endothelial growth factor receptor] receptor related). Cleft thorax is produced by mutant alleles of the JNK kinase (JNKK) hemipterous (*hep*) (30) or of the AP-1 subunit *kayak* (*kay*; the Drosophila ortholog of mammalian c-*Fos*) (31). The use of *pnr*-GAL4 or other drivers to bring about the local downregulation of JNK targets, such as *scarface* (serine protease) (32), or overexpression of the AP-1 target *puckered* (*puc*; a phosphatase regulator of JNK via a negative feedback loop) (33) or the *tissue inhibitor of metalloproteases* (*Timp*) (34) can also produce cleft thorax, while downregulation of *puc* can rescue cleft thorax caused by mutations of *hep* (30). One key target of JNK in dorsal closure (35, 36) is the transforming growth factor β family member decapentaplegic (dpp). In thoracic closure, *dpp* promotes the migration of cells at the imaginal leading edge (7), but it acts in a parallel pathway rather than downstream of JNK (30). One key target of *dpp* in thoracic closure is *pnr* (37). A *dpp* homologue in mammals is similarly involved in palatal closure (38).

We therefore set out to test whether AOX could rescue cleft thorax when induced by manipulations of the JNK pathway and the associated gene network described above.

## RESULTS

### AOX expression mitigates cleft thorax due to downregulation of JNK signaling.

**FIG 2:**
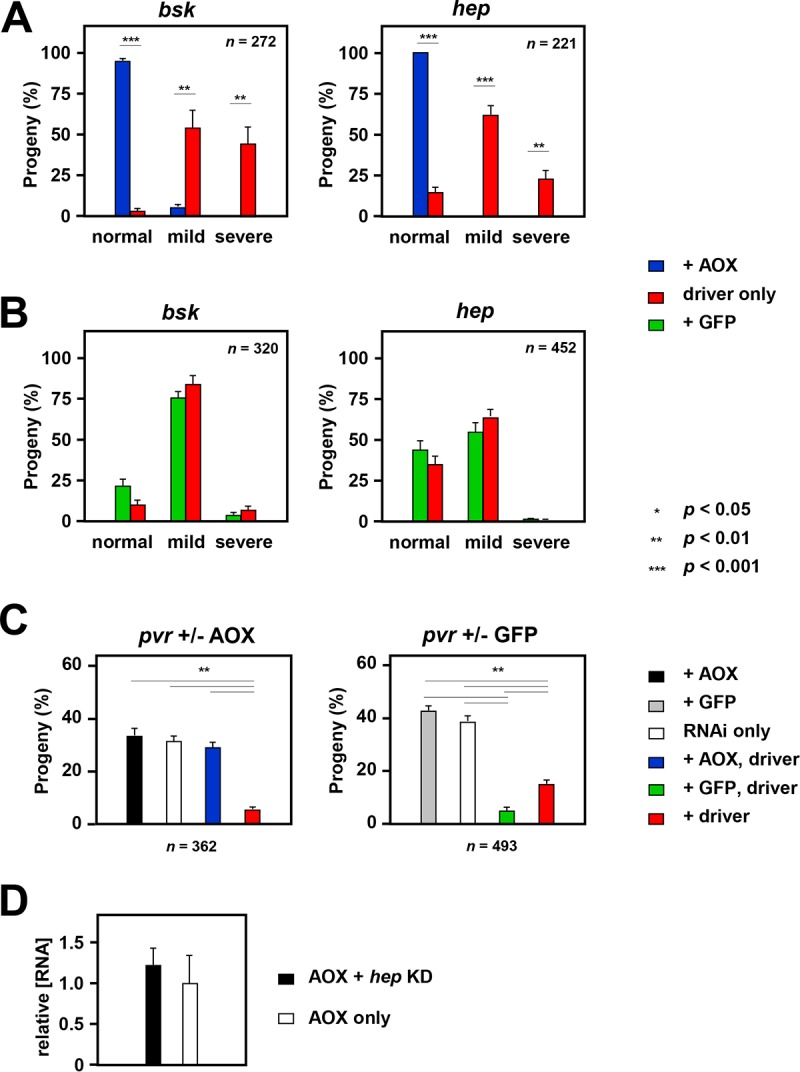
AOX rescues cleft thorax produced by downregulation of JNK signaling. (A, B) Effects of coexpressing AOX (A) or GFP (B) on the proportion of different phenotypic classes resulting from knockdown of *bsk* and *hep*, using the *pnr*-GAL4 driver and RNAi lines KK 104569 (*bsk*) and GD 47507 (*hep*). For details of the crosses, see Table S2 in the supplemental material. The data represent the means ± SEM for nine replicate vials in each experiment, with *n* indicating the total number of flies analyzed in each case. Statistically significant differences between the proportions of AOX- or GFP-expressing and -nonexpressing flies of different phenotypic classes are shown. *P* values, as indicated, were determined by paired, two-tailed Student's *t* test with Bonferroni correction. (C) Effect of coexpressing AOX or GFP on pupal semilethality caused by knockdown of *pvr* (RNAi line KK 105353) using the *pnr*-GAL4 driver. For details of the crosses, see Table S2 in the supplemental material. The data represent the means ± SEM for nine replicate vials in each experiment, with *n* indicating the total number of flies analyzed in each case. Statistically significant differences between classes are indicated, with *P* values being determined by analysis of variance with the Tukey *post hoc* honestly significant difference (HSD) test. Note that conversion to percentages for each vial corrects for differential lethality and for other vial-specific anomalies. (D) qRT-PCR analysis of AOX RNA (means ± SD; *n* = 3) in hemizygous *UAS-AOX^F6^* transgenic flies that were also hemizygous for *pnr^MD237^* (*pnr*-GAL4), with or without the *hep* RNAi construct of line GD 47509. Values were normalized against those for RpL32 and then against the mean value for flies expressing AOX only, to generate the relative values shown. KD, knockdown.

**FIG 3:**
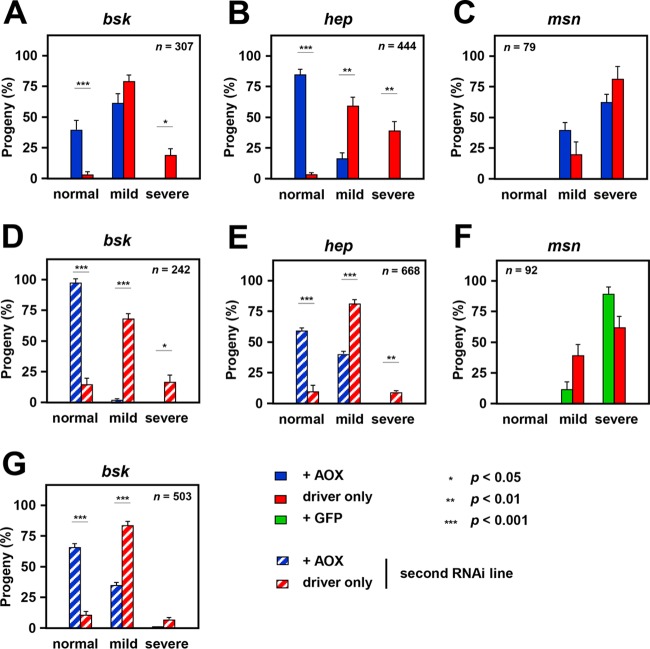
Confirmation of AOX rescue of cleft thorax caused by JNK knockdown. Effects of coexpressing AOX or GFP on the proportion of different phenotypic classes resulting from knockdown of *bsk*, *hep*, and *msn*, using the *pnr*-GAL4 driver. (A, B) Repeats of experiments whose results are shown in Fig. 2A. (C) Results of assays with RNAi line KK 101517 (*msn*) with coexpression of AOX. (D, E, G) Repeats of the experiments whose results are shown in Fig. 2A, using alternate RNAi lines, GD 38138 (*bsk*) and GD 47509 (*hep*). (F) Results of assays with RNAi line KK 101517 (*msn*) with coexpression of GFP. For details of the crosses, see Table S2 in the supplemental material. The data represent the means ± SEM for nine replicate vials in each experiment, with *n* indicating the total number of flies analyzed in each case. Statistically significant differences between the proportions of AOX- or GFP-expressing and -nonexpressing flies of different phenotypic classes are shown. *P* values, as indicated, were determined by paired, two-tailed Student's *t* test with Bonferroni correction. Note that conversion to percentages for each vial corrects for differential lethality and for other vial-specific anomalies.

We first confirmed, using RNA interference (RNAi) and the *pnr*-GAL4 (*pnr^MD237^*) driver, that the downregulation of key components of the JNK signaling cascade (Fig. 1A) (22) in the mediodorsal region during Drosophila development resulted in a phenotype of cleft thorax. After verifying the expression pattern conferred by *pnr*-GAL4, using the GFP reporter already present in the *pnr^MD237^* stock (Fig. 1B; see Table S1 in the supplemental material), we combined it with RNAi insertions targeted against *basket* (*bsk*; encoding JNK), hemipterous (*hep*; encoding JNKK), *misshapen* (*msn*; JNKKKK), and *PDGF- and VEGF-receptor related* (*pvr*; encoding the receptor tyrosine kinase at the top of the cascade [Table S2]). These all produced a cleft thorax phenotype of various severities (see Fig. 1C for examples), according to the tested construct/insertion and temperature. The two isolates of the *pnr*-GAL4 driver gave indistinguishable morphological phenotypes and were therefore used interchangeably in the remainder of the study. Under conditions producing the clearest phenotypes, but avoiding substantial lethality (except in the case of *pvr*, where it was unavoidable), we then combined these with expression constructs for AOX or for a control transgene, the GFP gene (Fig. 2 and 3).

For *bsk* and *hep* we tested multiple RNAi lines (Table S1), each producing cleft thorax when combined with the *pnr*-GAL4 driver. Although the severity of cleft thorax varied slightly between experiments, expression of AOX (Fig. 2A and 3A and B) but not that of GFP (Fig. 2B) led to a significant and substantial shift toward a wild-type phenotype in the progeny of *bsk* or *hep* knockdown flies. Two different RNAi lines for each gene showed the same effect (Fig. 3E to G). Any contribution to the alleviation of the phenotype from promoter dilution was excluded by measuring the amount of AOX RNA driven by *pnr*-GAL4 in pupae with and without one of the double-stranded RNA (dsRNA) constructs for *hep*, which showed no significant difference (Fig. 2D).

For *msn* knockdown, very few flies eclosed using the available RNAi line, and AOX or GFP expression produced no significant change in phenotype, despite a trend toward the wild type for AOX (Fig. 3C) and increased severity in the case of GFP (Fig. 3F). A *pvr* knockdown line was pupal semilethal when combined with the *pnr*-GAL4 driver, even at 18°C. As a result, the number of eclosing progeny was insufficient to enable a statistically meaningful analysis of the thoracic phenotype according to severity, but the mean proportion of progeny with cleft thorax was about 80% in this and parallel *pvr* knockdown experiments. Coexpression of AOX, but not GFP, gave substantial rescue of semilethality (Fig. 2C), with 71% (75/105) of the eclosing flies having a normal thorax.

### AOX expression can influence mammalian cell migration.

**FIG 4:**
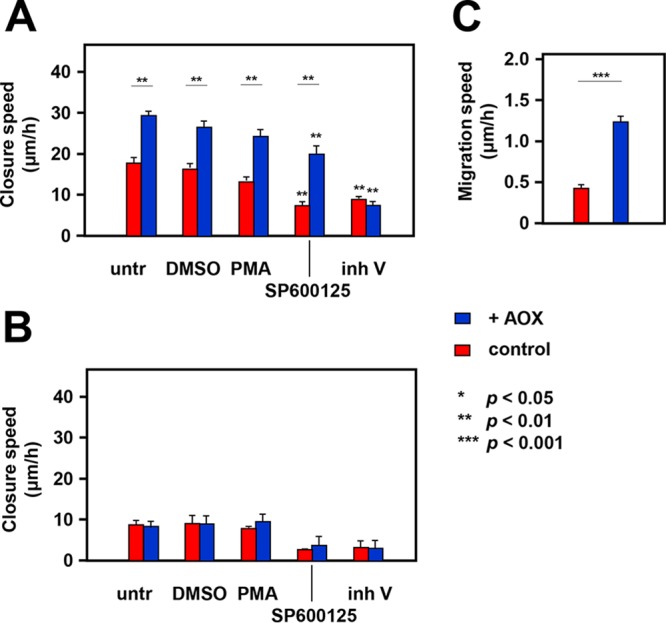
Effects of AOX on mammalian cell migration. (A, B) Rate of wound closure in scratch assay of cultured wild-type iMEFs (control) and AOX hemizygous iMEFs (A) and primary MEFs (B) at passage 6, as indicated, either untreated (untr), treated with only 0.2% DMSO, with PMA (20 mM), with SP600125 (20 μM in 0.2% DMSO), or with JNK inhibitor V (inh V; 20 μM), as shown. Asterisks above the bars indicate a statistically significant difference (determined by one-way analysis of variance with the Tukey *post hoc* HSD test) from untreated cells of the given genotype. Asterisks joining the bars indicate statistically significant differences between the genotypes for a given treatment, based on the same statistical analysis. For clarity, other significant differences are not shown. All data points are based on three biological replicates, each analyzed in triplicate, except for DMSO only, which used only two biological replicates. For the primary MEFs in panel B, the means ± SD are for pooled data from two cell lines of each genotype analyzed in triplicate at passage 6. (C) Rate of migration of single iMEFs of the indicated genotypes. Asterisks denote statistical significance, as shown (Student's *t* test, unpaired; *n* = 31 for control iMEFs; *n* = 21 for AOX-endowed iMEFs).

The failure of thoracic dorsal closure during Drosophila development indicates a defect in cell migration, which AOX expression was able to correct. To test the generality of this finding, we conducted cell migration assays in mammalian cells. Mouse embryonic fibroblasts (MEFs) were isolated from AOX hemizygous mice and wild-type littermates and immortalized using a standard retroviral transduction procedure with viruses encoding human papillomavirus 16 (HPV16) oncoproteins E6 and E7 (39). AOX-endowed immortalized MEFs (iMEFs) showed an increased speed of wound closure in the standard scratch assay (Fig. 4A), which was maintained in the presence of various drugs, notably, phorbol myristate acetate (PMA), an indirect activator of AP-1-dependent transcription (acting via protein kinase C), and the JNK inhibitor SP600125. However, JNK inhibitor V decreased the rate of wound closure of AOX-endowed iMEFs to the same level as wild-type iMEFs. The scratch assay conducted on primary MEFs (at passage 6) revealed no difference in migration rate between AOX-endowed and control MEFs (Fig. 4B). All primary lines migrated much more slowly than iMEFs, with inhibitor V also producing substantial cell death. The rate of single-cell migration of AOX-endowed iMEFs was also significantly greater than that of control iMEFs (Fig. 4C).

### AOX expression has no systematic effect on c-Jun phosphorylation.

**FIG 5:**
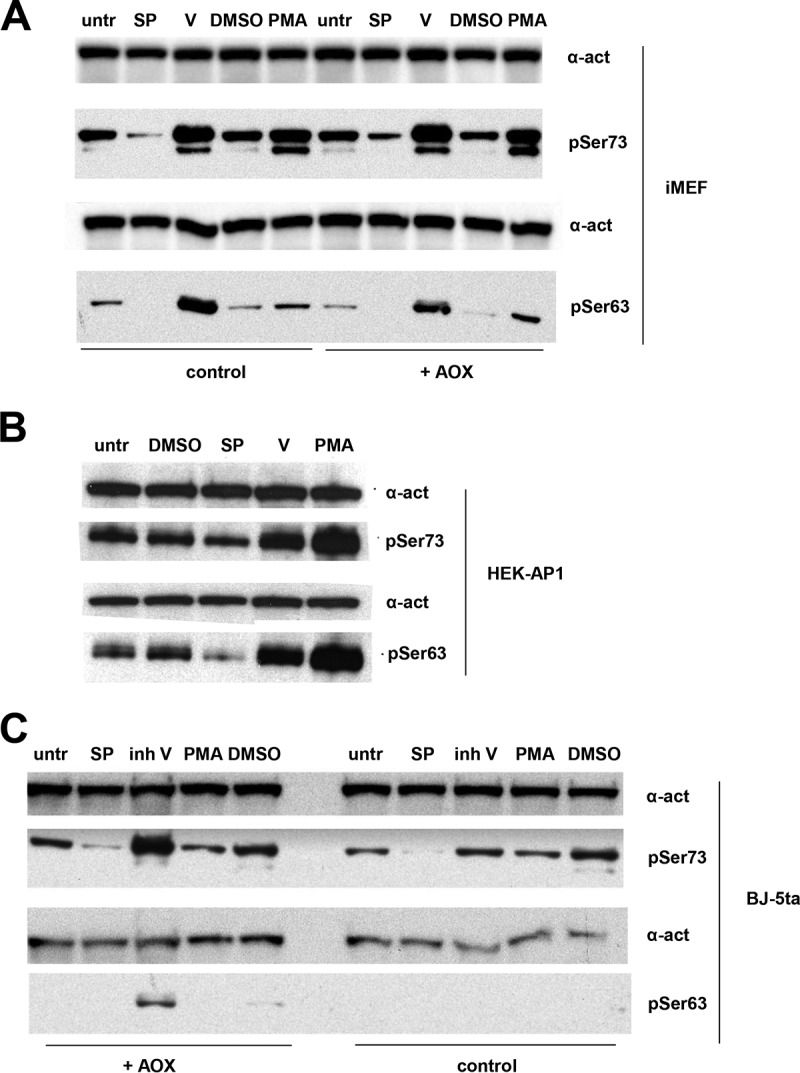
Phosphorylation status of JNK target residues in c-Jun. Western blots of whole-cell protein extracts from control and AOX-expressing iMEFs (A), HEK-AP1 cells (B), and AOX-expressing or control human BJ-5ta fibroblasts (C) untreated (untr) or treated with JNK modulators, as shown: 0.2% DMSO, 20 μM SP600125 (SP) in 0.2% DMSO, 20 μM JNK inhibitor V (V or inh V), or 8 nM PMA. The molecular weights of the major bands detected by each antibody, inferred from size markers run on all gels, were as expected (100 kDa for α-actinin [α-act], 47 kDa for c-Jun phosphorylated at residue Ser73 [pSer73] or Ser63 [pSer63]). Separate blots were initially probed for pSer73 or pSer63, and then in both cases the blots were reprobed for α-actinin as a loading control. Drug concentrations were based either on dose-response curves obtained using the HEK-AP1 cell transcriptional reporter system (for PMA and JNK inhibitor V; see Table S3 in the supplemental material) or on trials to determine the highest concentration at which there was no evidence of substantial cell death (for SP600125). Blot images were optimized for brightness and contrast, rotated, and cropped with the addition of white frames or dividers for clarity, but with no other manipulations.

We next tested the same set of JNK modulators for their effects on c-Jun phosphorylation at JNK target sites (40) Ser63 and Ser73, which has been shown to promote wound healing in the scratch assay (41). This was done in iMEFs (Fig. 5A), as well as in two other cell lines, the HEK293-derived AP-1 transcriptional reporter line used later in the study (HEK-AP1) (Fig. 5B) and human fibroblast line BJ-5ta (Fig. 5C). Only SP600125 decreased the amount of phosphorylated c-Jun, whereas JNK inhibitor V instead increased it, as did PMA. The presence of AOX did not influence c-Jun phosphorylation at these sites in iMEFs (Fig. 5A), although it did appear to potentiate the effect of inhibitor V in an AOX-expressing BJ-5ta cell clone (Fig. 5C).

### AOX does not rescue cleft thorax caused by manipulation of AP-1 expression or other targets.

**FIG 6:**
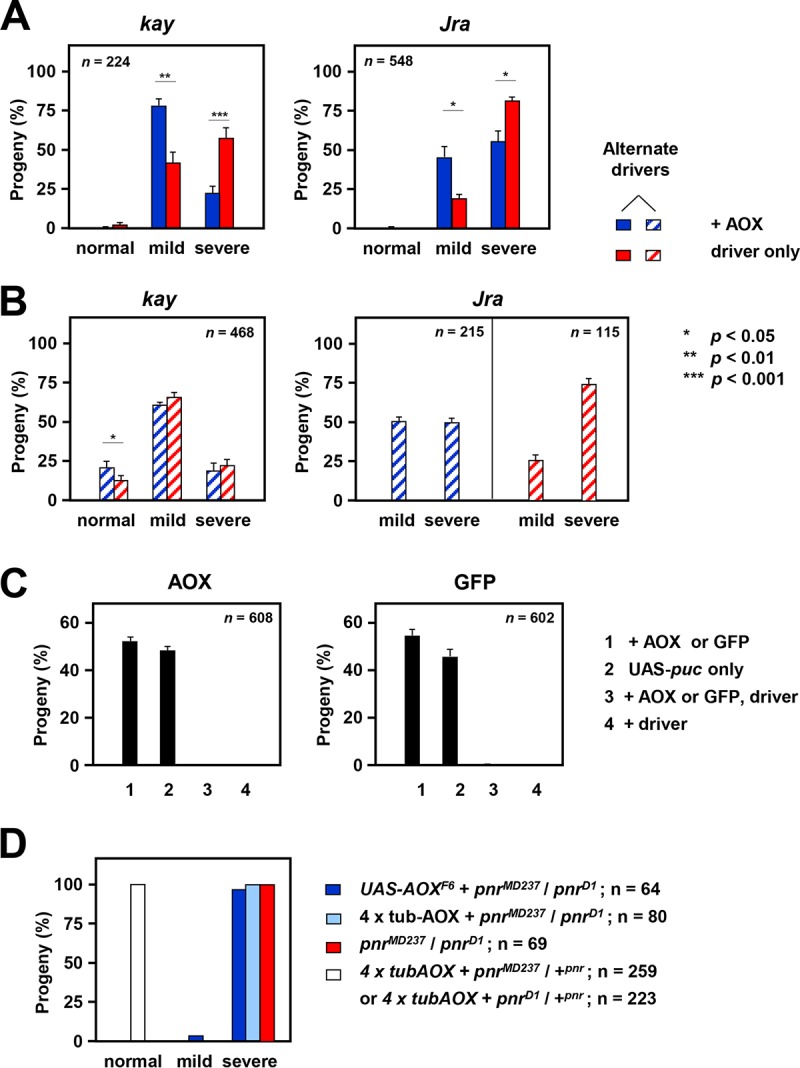
AOX does not rescue cleft thorax produced by altered expression of AP-1 or other targets. (A, B) Effects of coexpressing AOX on the proportion of different phenotypic classes resulting from knockdown of *kay* (at 25°C) and *Jra* (at 18°C), using the *pnr*-GAL4 driver and RNAi lines GD 6212 (*kay*) and KK 107997 (*Jra*) (A) and alternate RNAi lines GD 19512 (*kay*) and GD 10835 (*Jra*) (B). For details of the crosses, see Table S2 in the supplemental material. Because the GD 10835 (*Jra*) construct is carried on chromosome X, two parallel crosses were required to test the effects of AOX expression in each sex, and statistical analysis was not meaningful in this case. The data represent the means ± SEM for nine replicate vials in each experiment, with *n* indicating the total number of flies analyzed in each case. Statistically significant differences between the proportions of AOX-expressing and -nonexpressing flies of different phenotypic classes are shown. *P* values, as indicated, were determined by paired, two-tailed Student's *t* test with Bonferroni correction. Note that *Jra* knockdown using RNAi line KK 107997 (*Jra*) was lethal at 25°C and that AOX did not rescue this lethality. (C) Effect of coexpressing AOX or GFP on pupal lethality caused by overexpression of *puc* under the control of the *pnr*-GAL4 driver. Progeny classes are as indicated, and all contained, in addition, the UAS-*puc* overexpression construct. For details of the crosses, see Table S2 in the supplemental material. The data represent the means ± SEM for nine replicate vials in each experiment, with *n* indicating the total number of flies analyzed in each case. Note that conversion to percentages for each vial corrects for differential lethality and other vial-specific anomalies. (D) Phenotypes of *pnr^MD237^/pnr^D1^* compound heterozygotes with and without the presence of AOX transgenes, as indicated. Neither the GAL4-driven *UAS-AOX^F6^* transgene nor homozygosity for the *tub-AOX* transgenes on chromosomes 2 and X produced the rescue of the strong cleft thorax phenotype. Note that because progeny phenotypes were essentially uniform for a given genotype, no meaningful variances could be calculated.

We reasoned that directly downregulating c-Jun or its dimerization partner, c-Fos, encoded in Drosophila by *Jun-related antigen* (*Jra*) and *kayak* (*kay*), respectively, should produce effects that largely override its regulation by JNK and the beneficial effects of AOX. Accordingly, coexpression of AOX had only a slight effect on the severity of cleft thorax induced by knockdown of *kay* or *Jra* using the *pnr*-GAL4 driver (Fig. 6A and B). Similarly, AOX was unable to rescue the lethality caused by overexpression of the AP-1 target, *puc*, which also antagonizes the action of *bsk* (Fig. 6C). Note, however, that this result may be trivial, since *puc* overexpression generates a severe embryonic phenotype, due to the inhibition of dorsal closure.

As discussed earlier, *pnr* is considered to act in thoracic closure via a pathway parallel to the JNK pathway. Since the *pnr*-GAL4 line *pnr^MD237^* is also a *pnr* hypomorph, we combined it with the *pnr^D1^* mutant as a compound heterozygote, producing, as expected, a phenotype of severe cleft thorax (Fig. 6D). This was not alleviated by AOX, whether it was supplied using a constitutive or a GAL4-dependent transgene (Fig. 6D). Our findings are consistent with the inference that AOX acts on JNK signaling upstream of AP-1 but cannot compensate for a deficiency of AP-1 itself nor of a parallel pathway also required for thoracic closure.

### AOX does not influence AP1-dependent transcription in cultured cells.

To investigate the mechanism by which AOX impacts the outcome of JNK signaling, we tested whether it influences transcription directed by AP-1. Using a well-established luciferase-based AP-1 reporter system (42) and a variety of different expression constructs for AOX, we tested whether AOX expression in Drosophila S2 cells was able to alter AP-1-dependent transcription under different conditions of JNK pathway activation. First, we compared the transcriptional readout in cells cotransfected with the reporter plasmids and with AOX cloned into the copper-inducible expression vector pMT/V5-His B, with its natural stop codon, with that in cells transfected with the empty vector. JNK pathway activation was achieved using the pUAST-Hep^act^ plasmid, included in all transfections in combination with pAct-Gal4, which promotes pUAST-Hep^act^ transcription by constitutive expression of Gal4. Transfection efficiency was controlled by the inclusion of a constitutively expressed plasmid encoding renilla luciferase, which can be experimentally distinguished from the firefly luciferase of the reporter construct. Finally, to measure background transcription independently of AP-1, the system includes a mutated version of the reporter (which was used as an alternative in transfections), to which AP-1 does not bind.

**FIG 7:**
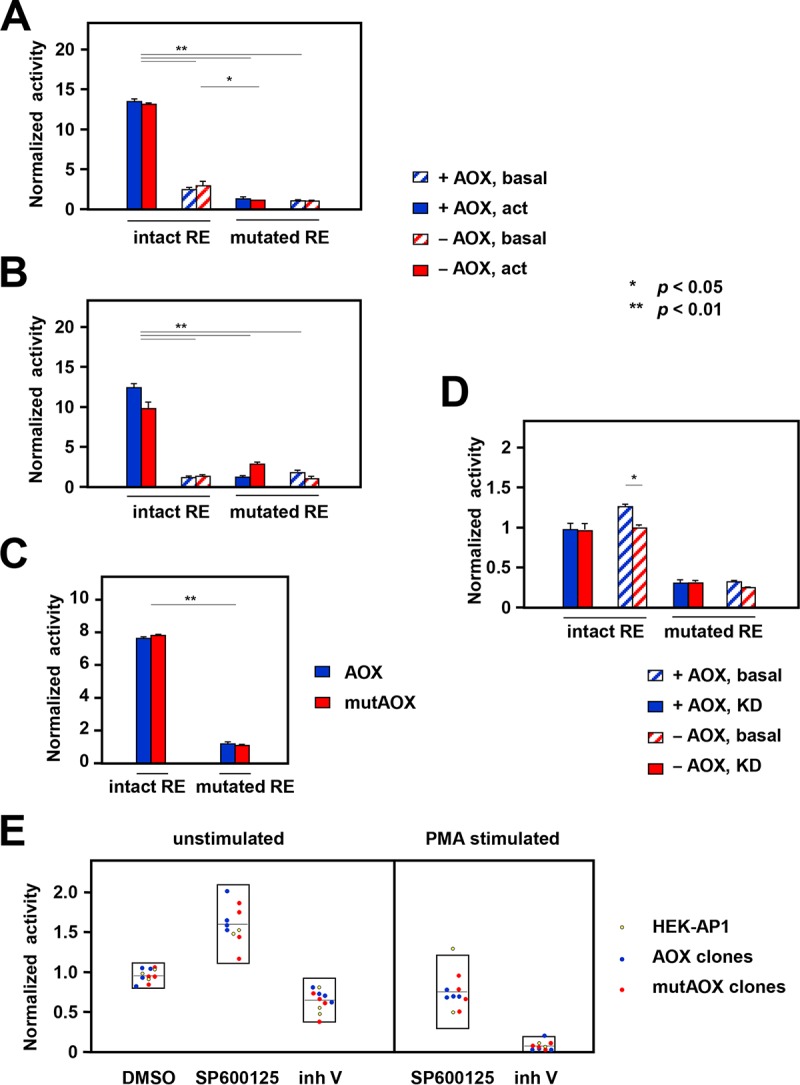
Effects of AOX on AP-1-dependent transcription. (A to D) Firefly luciferase activity in extracts of S2 cells cotransfected with pMT-AOX (or empty vector) (A), pAC/AOX (or empty vector) (B), or pUASTattB-AOX (or pUASTattB-mutAOX) (C), with pUAST-Hep^act^ together with pAct-Gal4 (or not) to activate it (A to C) or following 5 days of dsRNA treatment (knockdown) for *hep* (D), and with reporter constructs containing intact (TRE-fluc) or mutated (mRE-fluc) phorbol ester-response elements (RE), as indicated, and pAct-RL for normalization. All data were first normalized for transfection efficiency, based on renilla luciferase activity, followed by renormalization against basal activity for the relevant control cells, i.e., the empty vector (A, B, D) or pUASTattB-mutAOX (C). The results for AOX-expressing and AOX-nonexpressing cells in any category were not significantly different from each other (Student's *t* test, two-tailed, unpaired), apart from one case in panel D, as indicated. Statistically significant differences between data classes were determined by one-way analysis of variance with the Tukey *post hoc* HSD test; for clarity, only comparisons between the pairs which were coherent are shown, except in panel D, where some significance values differed, as shown. See also Table S4 in the supplemental material for the results of repeat and parallel experiments. act, activated. (E) Firefly luciferase activity in extracts of HEK-AP1 reporter cells and clones derived from them transduced with AOX- or mutAOX-expressing lentiviral constructs (see Table S5 for clone characterization) and treated with the indicated drugs: 0.2% DMSO, 20 μM SP600125 in 0.2% DMSO, or 20 μM JNK inhibitor V. Data were normalized against the values for the corresponding untreated cells (unstimulated) or for the corresponding cells stimulated with 8 nM PMA, as shown. Box plots indicate the means and the 95% confidence intervals for each data set.

Despite the complexity of this system, it gave clear-cut results. AOX produced no significant change in AP-1-dependent luciferase expression under both basal and JNK-activated conditions (Fig. 7A; Table S4). Next, we tested reporter cells cotransfected with a plasmid (pAC/AOX) (43) directing constitutive AOX expression under the control of a β-actin promoter versus cells cotransfected with the empty vector. Again, AOX expression had no effect on the transcriptional readout (Fig. 7B; Table S4). Using a system in which JNK pathway activation and AOX induction were brought about simultaneously by expression of the exogenous transcription factor Gal4, but this time using a control plasmid harboring a catalytically inactive, mutated AOX, we again found no effect of AOX (Fig. 7C: see also the results of a parallel experiment in Table S4). AOX also produced no significant difference in AP-1-dependent transcription in cells where *hep* had been knocked down (Fig. 7D; Table S4).

We conducted a similar exercise in mammalian cells, using an HEK293 cell-derived reporter cell line (here designated HEK-AP1), stably transduced with lentiviral constructs expressing AOX or, as a control, the mutated, catalytically inactive variant (mutAOX). Successful transduction and cell cloning at limiting dilution were verified via the fluorescence conferred by the cotransduced marker GFP, and AOX functionality was verified by respirometry (Table S5). Although individual HEK-AP1 cell-derived clones showed a variable degree of AP-1-dependent transcriptional activity, AOX-expressing and control cell clones showed a similar susceptibility to the effects of the JNK antagonists SP600125 and inhibitor V (Fig. 7E). Surprisingly, SP600125 increased rather than decreased the transcriptional readout, despite the fact that it inhibited c-Jun phosphorylation (Fig. 5B), although it did modestly suppress PMA-activated transcription in the reporter line (Fig. 7E).

### Antimycin A differentially stimulates the migration of AOX-expressing cells.

**FIG 8:**
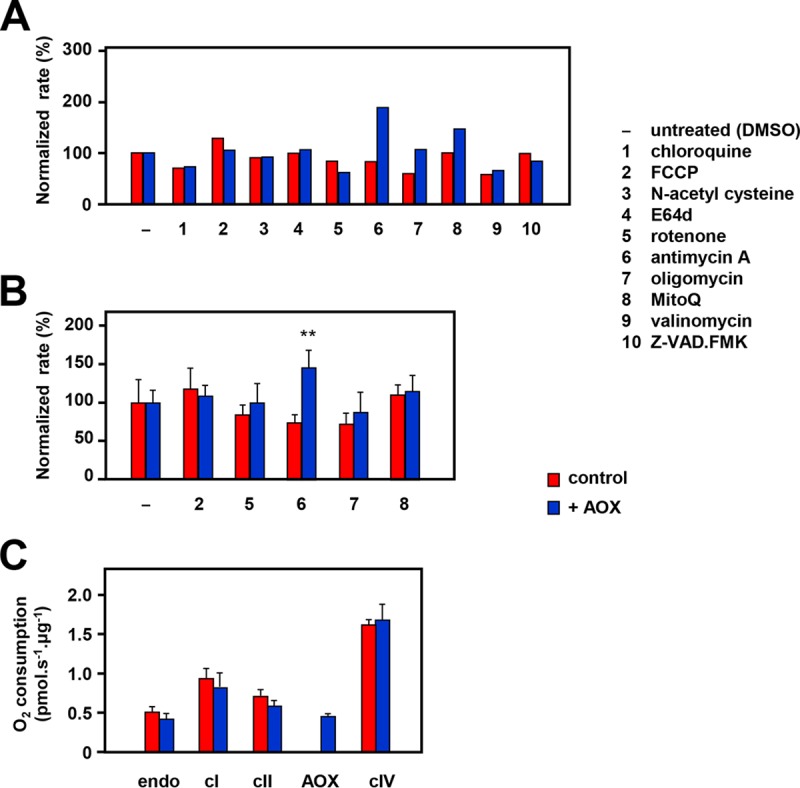
Modulation of iMEF migration by metabolic inhibitors. (A, B) Relative rate of wound closure for wild-type iMEFs (control) and AOX-hemizygous iMEFs treated either with DMSO only (—) or with the indicated drugs (see Materials and Methods). Data are normalized against the closure rate for the given cell line treated only with DMSO. (A) Preliminary experiment with 9 different drugs. (B) For the 5 drugs indicated, the data represent the means ± SD from 4 independent experiments. Asterisks indicate statistically significant differences (Student's *t* test, *P* < 0.01) between drug-treated and untreated (DMSO only) cells of the given genotype. (C) Respiratory oxygen consumption of control and AOX-expressing iMEFs is indicated as follows: endo, endogenous whole-cell respiration; cI, cII, cIV, and AOX, respiration of permeabilized cells driven by cI-, cII-, or cIV-linked substrate mixes or by cII-linked substrate mix in the presence of antimycin A (denoted AOX), respectively.

To gain insight into the intracellular process(es) underlying the enhanced migratory behavior of AOX-expressing cells, we tested the effects of sublethal doses of various metabolic effectors on the relative rates of migration of AOX-expressing versus control iMEFs. In an initial experiment (Fig. 8A), we tested various oxidative phosphorylation inhibitors, antioxidants, and protease inhibitors in the wound-healing assay for a differential effect on AOX-expressing cells. For further study, we selected three treatments that appeared to give a differential effect (antimycin A, oligomycin, and mitoquinone mesylate [MitoQ]), together with two that did not (rotenone and carbonyl cyanide *p*-trifluoromethoxyphenylhydrazone [FCCP]), and measured wound closure in four independent experiments. Antimycin A had a significantly different effect on the migration of AOX-expressing MEFs versus wild-type MEFs (Fig. 8B), stimulating the migration of the former but suppressing that of the latter, whereas MitoQ, rotenone, oligomycin, and FCCP had no significant effects. To understand the implications of these findings for the mechanism by which AOX promotes cell migration, we checked the effects of AOX expression on respiration in the cell lines tested (Fig. 8C). AOX had no significant effect on whole-cell respiration or on permeabilized cell respiration on cI-, cII-, and cIV-linked substrates. However, in the presence of antimycin A, it enabled almost 80% of the uninhibited respiration rate in permeabilized cells, driven by succinate. This capacity actually exceeded the measured rate of whole-cell respiration under uninhibited conditions, implying that the capacity for AOX-mediated respiration was sufficient to maintain normal respiratory electron flow in iMEFs in the presence of antimycin A.

## DISCUSSION

In this study, we showed that the expression of AOX is able to promote cell migratory behavior in two different models: in Drosophila, AOX expression corrected thoracic closure defects produced by impaired signaling at several steps in the JNK pathway (summarized in Fig. 1A), while AOX-expressing iMEFs (but not primary MEFs) showed enhanced migration, which was abolished by JNK inhibitor V. In contrast, AOX was unable to correct cell migration defects resulting from downregulation of the main JNK substrate, the c-Jun subunit of AP-1, or by manipulation of other factors, such as the AP-1 downstream target *puc* or the *pnr* transcription factor. Using a luciferase reporter system, AOX also had no effect on AP-1-dependent transcription in proliferating Drosophila (S2) or mammalian (HEK293-derived) cells. The migration of AOX-expressing iMEFs compared with that of control iMEFs was differentially stimulated by antimycin A but not by other drugs that affect mitochondrial electron flow, ATP production, reactive oxygen species (ROS), or membrane potential. We thus propose increased mitochondrial heat production as the most likely mechanism by which AOX promotes cell migration.

### Conditions for AOX activation.

Clearly, to affect cell motility or any other phenotype, AOX needs to be enzymatically active, modifying the cellular metabolic state. Previous studies in model systems (13–19) found that AOX expression has only minimal effects on phenotype under standard physiological conditions. Its activation requires that its substrate, ubiquinol, accumulate to substantial levels in the reduced form (44, 45), due to respiratory chain inhibition or overload. Other aspects of metazoan AOX regulation remain largely unknown. In plants, AOX is activated by pyruvate (44, 46), although this regulation has not been demonstrated in the metazoan enzyme (47). Nevertheless, AOX rescue of cleft thorax and promotion of iMEF motility imply that it can become activated at the margin of a migrating cell sheet. Cell movement is an energy-requiring process, and AMP-activated protein kinase (AMPK), one of the cell's key regulators of energy homeostasis (48), is locally activated at the leading edge of a migrating cell sheet (49), where it promotes the movement of mitochondria into this subcellular domain. This metabolic microenvironment may also favor AOX activation, if the extra demand exceeds the capacity of respiratory complex III. In addition to AMPK, the mitochondrial unfolded protein response (mtUPR), which has previously been linked with JNK signaling (50), may be elicited. Treatment with antimycin A, which was shown here to differentially stimulate the migratory behavior of AOX-expressing iMEFs, should lead to the further activation of AOX, since blockade of cIII increases the ratio of ubiquinol to ubiquinone, diverting electron flow through AOX.

The proteins used for immortalization of MEFs, namely, the HPV16 E6 and E7 oncoproteins, may also synergize with activated AOX. Among other effects (51), E7 switches energy supply from respiration to glycolysis by activating pyruvate kinase (52). Switching to the high-capacity glycolytic pathway may underlie the increased migratory capacity of immortalized MEFs, as inferred previously for cancer cells (53, 54). AOX may enhance this by increasing pyruvate clearance via the tricarboxylic acid cycle, promoting glycolytic flux. Similarly, E6 promotes glucose transport (55–57) and facilitates the degradation of p53 (58), providing a possible link to AP-1 and JNK (59–62). E6 also upregulates hypoxia-inducible transcription factor 1α (HIF-1α) (63) and mTORC1 (64).

### Is AP-1 the target of AOX?

Reporter assays indicate that AP-1 and its transcriptional activity are not a direct target of AOX, although the Drosophila findings and the effects of inhibitor V leave open a possible direct involvement of JNK, acting through other targets.

The transcriptional reporter cell assays were conducted in proliferating cells, where AP-1 may act differently than in a migrating cell sheet. It plays diverse physiological roles, and each of its subunits is encoded by a gene family (65). Although its canonical members, c-Fos and c-Jun, drive cell proliferation (66), others are functionally diverse and are regulated by a host of mechanisms (67), including subunit composition (68), other MAP kinases (69), and mitochondrial ATP depletion (70).

The interpretation of experiments using JNK inhibitors rests upon assumptions regarding their specificity, but the effects of SP600125 and JNK inhibitor V do not fit previous assumptions regarding their mode of action. Inhibitor V was the only drug tested which blocked the migration-promoting effects of AOX in iMEFs, indicating that it acts at a step different from that influenced by AOX. These drugs have previously been studied almost exclusively in highly artificial *in vitro* systems (49, 71), taking little account of their demonstrated activity against other kinases (71–73). The alternative approach of using genetic ablation of JNK to elucidate the AOX-responsive step cannot easily be applied in mammalian cells, since JNK has diverse isoforms created by alternative splicing and is encoded by a multigene family, some of whose members have undoubtedly been functionally redeployed during evolution (74).

AP-1 is subject to oxidative inactivation (65, 75), from which it is protected in Drosophila by the coactivator MBF1 (76), and the loss of MBF1 sensitizes animals to cleft thorax, if also exposed to H_2_O_2_. AOX decreases net ROS production from mitochondria (15, 77), which could compensate for the impaired upstream activation of AP-1, but, as discussed below, its effects on mammalian cell migration were not influenced by drugs that alter ROS (MitoQ, *N*-acetyl cysteine; Fig. 8).

### AOX and other signaling pathways.

AOX may also act through other pathways independently of or in conjunction with AP-1. For example, immortalized fibroblasts show an enhanced induction of HIF-1α under hypoxia (78), while AOX also blunts the hypoxia response (79). In cancer cells, mitochondrial ROS activates many transcription factors (80), and interactions between AMPK and AP-1 have been reported in cardiomyocytes (81) and Jurkat T cells (82). Mitochondrial energy status also governs calcium transport into mitochondria, which has major effects on cell migration (83).

Other transcription factors mediate mitochondrial responses to stress, notably, Nrf2 (84), ATF4 (85), and other AP-1 paralogs. Although stressors that activate AP-1 via JNK have only minimal effects on Nrf2- or ATF4-induced transcription (86), this does not exclude the possibility of the converse. JNK can be activated by mitochondrial ROS in ischemia-reperfusion (87) or by the mtUPR (50). JNK itself has also been reported to interact directly with mitochondria, triggering downstream effects (88).

### AOX stimulates migratory behavior by a mechanism connected to metabolism.

Of the metabolic inhibitors tested, only antimycin A preferentially stimulated the migratory behavior of AOX-expressing cells compared with control cells in the wound-healing assay. This result provides a strong clue as to the mechanism of action of AOX. The ineffective treatments included agents that should increase mitochondrial membrane potential (oligomycin [89]) or decrease it (FCCP [90], rotenone [91]), other treatments that inhibit mitochondrial ATP production (oligomycin, FCCP, and rotenone) or drugs that increase ROS production (oligomycin [92], rotenone [91, 93]) or dampen it (*N*-acetyl cysteine [94], MitoQ [95]) or that can have effects in either direction, depending on the dose (FCCP [96–98]). Agents that limit proteolytic turnover of cellular components in lysosomes (chloroquine [99]; E64d [100]) or by caspases [carbobenzoxy-valyl-alanyl-aspartyl-(*O*-methyl)-fluoromethylketone (Z-VAD-FMK) (101)] also appeared to have no differential effect on the migration of AOX-expressing cells. Not only did these other treatments fail to influence control and AOX-expressing cells differentially, but none of them produced substantial alterations to the rate of migration of control cells.

Since AOX-expressing iMEFs continue to respire when treated with antimycin A, most metabolites should be only minimally affected. The biggest difference should be in the rate of mitochondrial ATP production, when respiratory complexes III and IV are replaced by the non-proton-motive AOX. However, as indicated above, mitochondrial ATP production as such cannot be the crucial factor determining the rate of migration.

In the presence of antimycin A, the energy released by AOX in catalyzing quinol oxidation is instead converted to heat. We earlier showed that AOX-driven respiration in human cells was able to maintain the same mitochondrial temperature with less than half the amount of respiratory flux (102). In the iMEFs tested, the capacity for AOX-mediated respiration was sufficient to maintain respiratory electron flow in the presence of antimycin A at the same rate as in untreated cells (Fig. 8C). This implies that the cells should be able to maintain normal redox homeostasis in the presence of the drug with only a minimal metabolic disturbance, arising from the need to make more ATP through nonmitochondrial pathways. Maintenance of respiratory flux but in which flux is driven through AOX rather than cIII implies a pronounced thermogenic effect. Thus, we propose increased mitochondrial heat production as the most likely mechanism whereby AOX stimulates cell migration.

Fibroblast motility has long been known to be temperature dependent (103), and actin polymerization itself is highly affected by changes in temperature (104). The migration of mesenchymal stem cells is stimulated by increased expression of heat shock protein 90 (105), which impinges on various signaling pathways, possibly underlying the different effects of JNK inhibitors with poor selectivity. Heat shock protein 70 has also been shown to promote cell migration, by acting as a chaperone for the delivery of proteins needed by migrating cells to the leading edge (106). Even a transient heat shock can promote cell migration in cancer cell lines (107), which may (108) or may not (107) depend on heat shock transcription factor 1 (HSF1).

Together, these data strongly suggest that AOX activation is able to promote cell migration by raising the intracellular temperature, most likely in specific subcellular compartments. The compensatory effects on JNK signaling are thus implied to be indirect, accounting also for the fact that AOX was able to correct cleft thorax in a completely different model generated by deranged nuclear receptor signaling (8).

Further validation of this hypothesis will require the development of quantitatively reliable methods to measure the intracellular temperature in specific cell compartments *in situ*. It will require even more sophisticated technology to make such measurements *in vivo* in the Drosophila pupa. Alternatively, if some other metabolic effect of AOX-driven respiration is responsible, we would need to wait for metabolomic technologies to advance to the single-cell level (109) to identify plausible candidates, although novel *in situ* methods would be needed to take this to the subcellular level.

### Developmental context of mitochondrial effects on cell migration.

Insect metamorphosis is fuelled by stored nutrients accumulated during larval growth, principally, lipid which is metabolized in mitochondria (110). A drop in the ATP level due to nutritional limitations should activate AMPK, so as to refocus resources onto ATP production. However, because all protein kinases depend on ATP as a substrate, ATP deficiency should restrain other regulatory kinases and limit ATP-consuming developmental processes, such as cell migration. Assuming that JNK operates as one such pathway, its inappropriate downregulation in the dorsal thoracic epithelium under conditions where the ATP supply is adequate could restrict cell migration while other developmental processes are energized normally, resulting in the specific failure of midline closure.

Rapid wound healing is also important for limiting pathogen invasion. Pathogens may deplete the nutritional environment of a wound, potentially jeopardizing the processes that support tissue repair. Rapid and efficient wound closure in a nutritionally limited environment may therefore depend on metabolic remodeling to support motility (111).

A full elucidation of the processes that link mitochondrial perturbations with cell migration should be of considerable medical importance and might even enable the design of new and more effective treatments, e.g., for metastatic tumors, tissue injuries, and congenital midline closure defects. Confirmation of a role in these processes for mitochondrial heat production may open up entirely new avenues for therapy.

## MATERIALS AND METHODS

### Drosophila strains and culture.

The Drosophila strains used in the study and their sources are summarized in Table S1 in the supplemental material. Flies were maintained in standard high-sugar medium (15) on a 12-h light/12-h dark cycle at 25°C, except where indicated in the figure legends. Crosses were generally implemented in triplicate, with flies being tipped into new vials on three successive days after mating.

### Cell culture.

Drosophila strain S2 cells were maintained as described previously (112). AOX-positive and -negative mouse embryonic fibroblasts (MEFs) (113), sourced either from embryos transgenic for C. intestinalis AOX, inserted at the *Rosa26* locus (18), or from their nontransgenic littermates, were studied at passage 6. MEFs were immortalized (iMEFs) by retroviral transduction with HPV16 oncoproteins E6 and E7 (39) and maintained at 37°C in 5% CO_2_ in Dulbecco modified Eagle medium (DMEM; Sigma-Aldrich) supplemented with 20% (primary MEFs) or 10% (iMEFs) heat-inactivated fetal bovine serum (FBS; Sigma-Aldrich), 1% penicillin-streptomycin (Lonza), and 4 mM l-glutamine (Sigma-Aldrich). An AP-1 reporter HEK293 recombinant cell line (JNK signaling pathway; BPS Bioscience), here abbreviated HEK-AP1, was maintained at 37°C in 5% CO_2_ in minimal essential medium (HyClone) supplemented with 10% heat-inactivated FBS (Sigma-Aldrich), 1% nonessential amino acids (HyClone), 1 mM sodium pyruvate (Sigma-Aldrich), 1% penicillin-streptomycin (Lonza), and 400 μg/ml Geneticin (Gibco, Life Technologies). Geneticin was freshly distributed to each plate when cells were passaged. BJ-5ta human fibroblasts (ATCC CRL-4001) were grown at 37°C in 5% CO_2_ in DMEM (Sigma-Aldrich) supplemented with 10% heat-inactivated FBS (Sigma-Aldrich), 20% medium 199 (Sigma-Aldrich), 1% penicillin-streptomycin (Lonza), and 4 mM l-glutamine (Sigma-Aldrich). HEK-AP1 and BJ-5ta cells expressing C. intestinalis AOX or the mutated, catalytically inactive variant mutAOX (43) were obtained by pWPI-based lentiviral transduction as described previously (114). Transduced cell populations were sorted according to GFP fluorescence using a BD FACSAria II cell sorter equipped with an 85-μm nozzle and operated at a sheath pressure of 45 lb/in^2^ and then cloned at limiting dilution. Clones were reverified for GFP fluorescence by fluorescence-activated cell sorting and for AOX functionality by respirometry, as described previously (43).

### Wound-healing and single-cell migration assays.

Samples of 90,000 cells were plated on 24-well plates (CellStar; Greiner Bio-One) as technical triplicates (3 wells per sample). After 24 h, cells were treated with one of the following reagents: 0.2% dimethyl sulfoxide (DMSO; Hybri-Max; Sigma-Aldrich), 20 μM SP600125 (Sigma-Aldrich) in 0.2% DMSO, 20 μM JNK inhibitor V (Merck), or 20 nM phorbol myristate acetate (PMA; Sigma-Aldrich) in fresh medium. After 2 h, a linear scratch was made in the center of each well with a p10 (1- to 10-μl) pipette tip. The cells were then washed three times with medium to remove detached cells, and fresh medium containing the appropriate reagent was added to each well. For testing the effects of metabolic inhibitors in this assay, cells were seeded at 45,000 to 90,000 cells per well on 24-well plates until a monolayer was formed (24 to 48 h), before the scratch was made. After rinsing once with normal medium, medium was replaced with 1 ml of fresh medium containing one of the following drugs: oligomycin (1 ng/ml), antimycin A (60 ng/ml), rotenone (150 nM), FCCP (10 μM), *N*-acetyl cysteine (5 mM), chloroquine (20 μM), E64d (10 μg/ml), MitoQ (250 nM; a kind gift of Mike Murphy, MRC Mitochondrial Biology Unit, Cambridge, UK), valinomycin (10 μM), Z-VAD-FMK (20 μM), or just 4 μl DMSO as a solvent control. All chemicals were from Sigma-Aldrich, except where stated otherwise. To measure single-cell migration, samples of 15,000 cells were plated on 6-well plates (CellStar; Greiner Bio-One) as technical triplicates (3 wells per sample). To minimize plate-specific effects, each plate contained both AOX-positive and -negative iMEFs in all assays. Cells were imaged as described below.

### Microscopy.

Imaging of fly thoraxes used a Nikon digital DS-Fi1 high-definition color camera with a Nikon SMZ 745T stereoscopic zoom microscope operated by NIS-Elements D (version 4.20) software. For live imaging of Drosophila embryos, eggs were collected from grape juice-agar plates after adult flies were placed in a mating chamber for 1 h, covered by a dark box, dechorionated using double-sided tape (115), placed into 35-mm glass-bottom microwell dishes (MatTek Corporation) filled with Halocarbon Oil 700 (Sigma-Aldrich), and imaged for GFP fluorescence using an Andor spinning disc confocal microscope equipped with an Andor Neo 5.5 sCMOS vacuum-cooled camera at ×20 magnification. The larvae were cleaned of adherent food using a soft paintbrush, dried on a precision wipe, placed on an ice-cold microscope slide (Menzel Gläser), and covered with a few drops of 50% ice-cold glycerol and then with a cover glass (thickness number, 1½; Zeiss). Samples were placed in a −20°C freezer for 15 min to immobilize the larvae. Live imaging used a Zeiss Axio Imager M2 upright microscope with a Zeiss EC Plan-Neofluar 5×/0.16, WD 18.5-mm air objective and ZEN software without the ApoTome function. Pupae were gently detached from the vial, placed with the dorsal side down into 35-mm glass-bottom microwell dishes (MatTek Corporation), and imaged as described above for embryos. Wound closure time-lapse images were taken with a ChipMan Technologies Cell-IQ observation incubator equipped with a Retiga EXi 1392 charge-coupled-device camera, using a Nikon CFI Plan Fluorescence DL objective (magnification, ×10) until the wound closed (approximately 48 h) or for just 24 h when metabolic inhibitor drugs were applied. Images were taken every 30 min. The incubator environment was held at a temperature of 37°C and had an atmosphere of 5% CO_2_, 19% O_2_, and 76% N_2_. Wound-healing analysis was performed using a Cell-IQ analyzer (version 4.3) by manually tracking the gap area, based on lines drawn along the wounded area of an image taken every second hour. The speed of the collective motion of the cells was measured in the most linear part of the wound area over time. In the experiment using metabolic inhibitors, the most linear time interval for closure was also analyzed and applied across all treatments and plates. To generate images for measuring single-cell migration, cells were transferred 24 h after plating to a Nikon BioStation CT instrument equipped with a Nikon DS-1QM camera and imaged at ×4 magnification. The environment in the incubator was held at a temperature of 37°C with a relative humidity of 85% and a 5% CO_2_ atmosphere, as described above for the wound-healing assay. Images were taken every 6 min, and movies were made using CL Quant (version 3.10) software. Movement was analyzed using CellTracker image-processing software with semiautomated migration detection (116), ignoring cells that were dying or dividing.

### qRT-PCR.

RNA was extracted from S2 cells (117), and quantitative reverse transcription-PCR (qRT-PCR) was performed (16) as described previously, using RpL32 mRNA as an internal standard. Primers for RpL32 and AOX were those used previously (16), and those for *hep* mRNA were TTGGTTTCTTGGGGTCGATG and TGGACTCCAAGGCCAACACT, the sequences of both of which are shown 5′ to 3′.

### Transfections and luciferase reporter assays in S2 cells.

Firefly/renilla luciferase dual-reporter assays in S2 cells used a cotransfection protocol (42), based on plasmids TRE-fluc, mRE-fluc, pAct-RL, pAct-Gal4, and pUAST-Hep^act^, kindly supplied by Dirk Bohmann, University of Rochester. Transfections used either TRE-fluc (a firefly luciferase reporter with the intact phorbol-ester response element) or mRE-fluc (with the mutated response element) plus pAct-RL (renilla luciferase for transfection normalization) with or without pAct-Gal4 together with pUAST-Hep^act^ for activated transcription and with the following plasmids or controls harboring AOX, as indicated in the figure legends: pMT-AOX (a kind gift of Filippo Scialó, Newcastle University), containing the C. intestinalis AOX-coding sequence recloned with its natural stop codon from the original vector, pCDNA5/FRT/TO (13), as an EcoRI fragment into the copper-inducible vector pMT/V5-His B (Thermo Fisher Scientific); pAC/AOX and pAC/mutAOX (43, 112), containing, respectively, the wild-type C. intestinalis AOX-coding sequence and the mutated, catalytically inactive variant under the control of the constitutive actin-5C promoter plus the corresponding empty vector pAc5.1/V5-His B (Invitrogen); and pUASTattB-AOX and pUASTattB-mutAOX (43), containing the same transgenes cloned into the pUASTattB vector under the control of a Gal4-dependent promoter. Expression and induction of AOX were verified for each plasmid by transfection and Western blotting as described previously (43), prior to use in luciferase reporter assays. Transfections used the FuGENE HD transfection reagent (Promega), according to the manufacturer's instructions, at a ratio of 3 μl FuGENE per μg of DNA. For each transfection, 3 × 10^5^ cells/ml were plated on 6- or 12-well plates. To each well was added a transfection mix consisting of FuGENE, 1 μg of each plasmid to be used (per ml of culture medium), and sterile water up to a final volume of 100 μl (6-well plates) or 50 μl (12-well plates). Transfections were carried out 1 h after plating (24 h in the case of pMT-AOX or the corresponding empty vector, with addition of 500 μM CuSO_4_ after a further 24 h), and luciferase assays were performed 72 h after transfection. In transfection mixtures to which mitochondrial inhibitors were added, 6 × 10^5^ cells/ml were plated on 6-well plates, and drugs were added 24 h after transfection at the concentrations shown in the figure legends. For luciferase reporter experiments in which *hep* expression was knocked down by RNAi, cells were also transfected with a dsRNA against the *hep* coding sequence, prepared by a two-round PCR-based procedure essentially as described previously (112), but using *hep*-specific primers (shown 5′ to 3′) TGGAGGCAAAGCTCCAGGC and CGCGAACGAAGCAGCCAAGG for the first round and GAATTAATACGACTCACTATAGGGGAGACATCCGCCACCCACGCACCTTC and GAATTAATACGACTCACTATAGGGGAGATCCCATTGCCCAGGTCGCCCAG for the second round, followed by transcription using a MEGAscript T7 transcription kit (Life Technologies). Knockdown at the RNA level (to ∼85%) was verified in transfected cells (112) by qRT-PCR. For combined dsRNA/reporter plasmid transfections, 1 × 10^5^ cells were plated per well in 24-well plates. After 30 min, cells were transfected with 300 ng of each relevant plasmid and 4 μg of *hep*-specific dsRNA per well in a total volume of 100 μl. A further 4 μg of the dsRNA was added 72 h later, and luciferase assays were conducted 110 h after the initial transfection. For luciferase assays, 75 μl of suspended cells from each well was transferred in triplicate to the wells of a 96-well microplate (Lab Systems) and analyzed using a Dual-Glo luciferase assay system (Promega), according to the manufacturer's protocol. Luminescence was measured using a Thermo Labsystems Luminoskan Ascent plate reader.

### Luciferase reporter assays in mammalian cells.

Firefly luciferase reporter assays were carried out in HEK-AP1 cells and in AOX/mutAOX-expressing clones derived from them, as follows: 30,000 cells were plated in technical duplicate (2 wells per sample) in luminometer-compatible Nunc MicroWell 96-well plates with lids (Thermo Fisher Scientific). After 24 h, the medium was replaced with medium containing either 0.2% DMSO, 20 μM SP600125 in 0.2% DMSO, 20 μM JNK inhibitor V, or no added drug. Cells were incubated for 2 h at 37°C. For PMA treatment, a second replacement medium contained 8 nM PMA plus 20 μM SP600125 in 0.2% DMSO, 20 μM JNK inhibitor V, or no other added drug, as appropriate, and the cells were incubated for a further 6 h. Luciferase assays were carried out using the Dual-Glo luciferase assay system (Promega), according to the manufacturer's protocol, and luminescence was measured using a PerkinElmer UV/visible plate reader.

### Protein analysis by Western blotting.

Batches of 300,000 MEFs or HEK-AP1 cells or 250,000 BJ-5ta cells were plated on 6-well plates (CellStar; Greiner Bio-One). After 24 h, the medium was replaced with medium containing either 0.2% DMSO, 20 μM SP600125 in 0.2% DMSO, 20 μM JNK inhibitor V, 8 nM PMA, or no added drug and the plate was incubated for 2 h (or 40 min, in the case of PMA). Cells were carefully rinsed in ice-cold phosphate-buffered saline (PBS) and then scraped free on ice using a CytOne cell scraper (220 mm long, 11-mm blade) in 75 μl of resuspension buffer containing 100 mM NaCl, 10 mM Tris-HCl, and 1 mM EDTA, pH 7.8, supplemented with cOmplete, Mini, EDTA-free protease inhibitor and phosphatase inhibitor cocktails (at the manufacturer's recommended amount; Roche) and 1 mM phenylmethylsulfonyl fluoride. Protein concentrations were determined using the Bradford assay. After lysis by the addition of an equal volume of SDS sample buffer (Laemmli 2× concentrate; Sigma-Aldrich), samples were heated for 5 min at 100°C and briefly centrifuged to remove particulates, and 20 μg of each extract was loaded onto 18-well precast Any kD Criterion TGX Stain-Free protein gels (Bio-Rad), which were run and blotted as described previously (43). Blots were processed as described previously (15), but with blocking in 5% bovine serum albumin (BSA) in PBS-Tween for 1 h on a shaker and using the primary antibody phospho-c-Jun (Ser 73) rabbit monoclonal no. 3270 (1:1,000; Cell Signaling Technology) or phospho-c-Jun (Ser63) II rabbit polyclonal 9261 (1:1,000; Cell Signaling Technology), with reprobing using anti-α-actinin rabbit polyclonal C-20 (1:7,000; sc-7454-R; Santa Cruz Biotechnology). Secondary antibody was peroxidase-labeled goat anti-rabbit IgG (1:10,000; PI-1000; Vector Laboratories). The chemiluminescence of all blots was documented both with film and by using a Bio-Rad ChemiDoc imager.

### Respirometry.

Whole-cell and permeabilized cell respiration was measured essentially as described previously (118). iMEFs were seeded 24 h before the experiment and grown in DMEM containing 4.5 g/liter glucose, 10% fetal bovine serum (Thermo Fisher Scientific), 2 mM GlutaMAX (Gibco), and 100 U/ml penicillin plus 100 μg/ml streptomycin (Lonza). To activate cell respiration, the growth medium was replaced 1 h before the assay. Cells were detached with 0.05% trypsin and counted by trypan blue exclusion. Mitochondrial respiration in permeabilized cells was assayed using an Oroboros oxygraph-2K oxygraph (Oroboros, Innsbruck, Austria), with 2 × 10^6^ iMEFs being directly suspended in the oxygraph chamber containing 2 ml of respiration buffer B (10 mM KH_2_PO_4_, 20 mM HEPES-KOH, 20 mM taurine, 0.5 mM EGTA, 3 mM MgCl_2_, 1 mg/ml essentially fatty acid-free BSA, 60 mM potassium-lactobionate, 110 mM mannitol, 0.3 mM dithiothreitol, pH 7.1). After measuring endogenous whole-cell respiration, substrates and inhibitors were added in the following order: (i) digitonin (30 μg), to permeabilize the cells; (ii) sodium pyruvate (to 5 mM), sodium glutamate (to 5 mM), and sodium malate (to 2 mM) as a cI-linked substrate mix, followed by ADP (to 2 mM); (iii) rotenone (to 150 nM) followed by succinate (to 10 mM) as a cII-linked substrate mix; (iv) antimycin A (to 30 ng/ml), to reveal AOX-mediated respiration; (v) *n*-propyl gallate (nPG; to 200 μM), to reveal any residual non-AOX-mediated oxygen consumption to be subtracted; (vi) *N*,*N*,*N*′,*N*′-tetramethyl-*p*-phenylenediamine (TMPD; to 1 mM) plus sodium l-ascorbate (to 2 mM) as a cIV-linked substrate mix; and (vii) sodium azide (to 40 mM), to reveal any non-cIV-mediated oxygen consumption to be subtracted. O_2_ consumption (in picomoles · second^−1^ · milliliter^−1^) was normalized to the amount of total proteins extracted from 1 × 10^6^ cells and assayed by the Bradford method (119). All chemicals were purchased from Sigma-Aldrich.

## Supplementary Material

Supplemental file 1

Supplemental file 2

Supplemental file 3

Supplemental file 4

Supplemental file 5
